# Time Course and Magnitude of Tolerance to the Ergogenic Effect of Caffeine on the Second Ventilatory Threshold

**DOI:** 10.3390/life10120343

**Published:** 2020-12-10

**Authors:** Carlos Ruiz-Moreno, Beatriz Lara, Jorge Gutiérrez-Hellín, Jaime González-García, Juan Del Coso

**Affiliations:** 1 Exercise Physiology Laboratory, Camilo José Cela University, 28692 Villanueva de la Cañada, Spain; cruizm@ucjc.edu (C.R.-M.); blara@ucjc.edu (B.L.); jgonzalez@ucjc.edu (J.G.-G.); 2 Faculty of Health Sciences, Francisco de Vitoria University, 28223 Pozuelo de Alarcón, Spain; jorge.gutierrez@ufv.es; 3 Centre for Sport Studies, Rey Juan Carlos University, 28943 Fuenlabrada, Spain

**Keywords:** exercise performance, sport performance, endurance performance, nutrition, endurance athlete

## Abstract

Pre-exercise caffeine ingestion has been shown to increase the workload at ventilatory threshold, suggesting an ergogenic effect of this stimulant on submaximal aerobic exercise. However, the time course of tolerance to the effect of caffeine on ventilatory threshold is unknown. This study aimed to determine the evolution of tolerance to the ergogenic effect of caffeine on the ventilatory threshold. Methods: Eleven participants (age 32.3 ± 4.9 yrs, height 171 ± 8 cm, body mass 66.6 ± 13.6 kg, VO_2max_ = 48.0 ± 3.8 mL/kg/min) took part in a longitudinal, double-blind, placebo-controlled, randomized, crossover experimental design. Each participant took part in two identical treatments: in one treatment, participants ingested a capsule containing 3 mg of caffeine per kg of body mass per day (mg/kg/day) for twenty consecutive days; in the other treatment, participants ingested a capsule filled with a placebo for the same duration and frequency. During these treatments, participants performed a maximal ramp test on a cycle ergometer three times per week and the second ventilatory threshold (VT_2_) was assessed by using the ventilatory equivalents for oxygen and carbon dioxide. Results: A two-way ANOVA with repeated measures (substance × time) revealed statistically significant main effects of caffeine (*p* < 0.01) and time (*p* = 0.04) on the wattage obtained at VT_2_, although there was no interaction (*p* = 0.09). In comparison to the placebo, caffeine increased the workload at VT_2_ on days 1, 4, 6 and 15 of ingestion (*p* < 0.05). The size of the ergogenic effect of caffeine over the placebo on the workload at VT_2_ was progressively reduced with the duration of the treatment. In addition, there were main effects of caffeine (*p* = 0.03) and time (*p* = 0.16) on VO_2_ obtained at VT_2_, with no interaction (*p* = 0.49). Specifically, caffeine increased oxygen uptake at VT_2_ on days 1 and 4 (*p* < 0.05), with no other caffeine–placebo differences afterwards. For heart rate obtained at VT_2_, there was a main effect of substance (*p* < 0.01), while the overall effect of time (*p* = 0.13) and the interaction (*p* = 0.22) did not reach statistical significance. Heart rate at VT_2_ was higher with caffeine than with the placebo on days 1 and 4 (*p* < 0.05). The size of the effect of caffeine on VO_2_ and heart at VT_2_ tended to decline over time. Conclusion: Pre-exercise intake of 3 mg/kg/day of caffeine for twenty days enhanced the wattage obtained at VT_2_ during cycling ramp tests for ~15 days of ingestion, while there was a progressive attenuation of the size of the ergogenic effect of caffeine on this performance variable. Therefore, habituation to caffeine through daily ingestion may reduce the ergogenic effect of this stimulant on aerobic exercise of submaximal intensity.

## 1. Introduction

Caffeine (1,3,7-trimethylxanthine) is a substance naturally found in coffee, tea, yerba mate and cocoa and is also added to foods and beverages due to its potent effect to increase alertness and to reduce fatigue. As a result, caffeine has become the most widely consumed psychostimulant in the world [1]. Caffeine also has a strong performance-enhancing effect [2] and it is widely used by athletes of numerous sport disciplines before and during competitions to enhance their physical performance [3]. In recent years, there has been a strong body of evidence, based on systematic reviews and meta-analyses, pointing towards an ergogenic effect of caffeine during endurance-like exercise activities [4,5,6,7] when caffeine is taken acutely in moderate doses (from 3 to 9 mg of caffeine per kg of body mass). These studies confirm the utility of acute caffeine intake to enhance the time to fatigue [8] and the mean power output during prolonged exercise trials [6], and the reduction in the time employed to complete a fixed amount of work during time trials [9]. The performance-enhancing effect of acute caffeine intake on endurance exercise may be associated to the effect of this substance to enhance maximal oxygen uptake [10], the exercise intensity at the anaerobic threshold [11,12] or the use of fat as a fuel, enabling higher muscle glycogen available late in exercise [13,14]. However, little is known about the magnitude of the ergogenic effect of caffeine on endurance performance when the substance is ingested chronically. Thus, to date, it is unknown if those endurance athletes consuming caffeine daily are benefiting from this substance during training and competition. 

The main physiological mechanism behind the performance-enhancing effect of caffeine on endurance exercise is associated to caffeine’s capacity to block A_1_, A_2a_ and A_2b_ adenosine receptors on the central nervous system [15], although other local effects have also been proposed as contributing mechanisms [16,17]. The blockade of adenosine receptors with caffeine intake, confirmed in both animals [18] and humans [19], is based on three different characteristics of caffeine when ingested orally: rapid absorption in the gut [20], its capacity to pass through all biological membranes, including the blood–brain barrier, due to its lipophilic nature [21] and its structural similarity to adenosine [22]. There, after acute caffeine ingestion and its posterior distribution through different tissues, caffeine hinders adenosine-specific receptors. This blockade eliminates, in part, the fatiguing effect of adenosine on the central nervous system, which explains the benefits for enhanced endurance performance. Interestingly, the ergogenic effect of acute caffeine intake increases with the duration of the exercise activity, suggesting that caffeine may better inhibit the negative effect of adenosine of physical performance as the exercise duration increases [4]. However, Fredholm [23] reported a growth in the number of adenosine receptors in the brain cortex of rats when these animals received caffeine for two weeks. Therefore, chronic intake of caffeine causes an increase in the number of adenosine-biding sites, which may reduce the efficacy of caffeine to block the deleterious effects of adenosine during exercise. This investigation led to the hypothesis that caffeine’s ergogenicity diminishes along with chronic ingestion, but evidence in humans is contradictory. 

When comparing the ergogenic response to caffeine in individuals with low/no daily caffeine intake versus individuals habituated to caffeine, it has been found that habituated individuals respond to caffeine at a similar magnitude as their unhabituated counterparts [24,25,26]. Although this finding contradicts the existence of tolerance to the ergogenic effect of caffeine, other researchers have found that unhabituated individuals responded better to caffeine than habituated individuals [27], while individuals with high chronic intakes of caffeine obtain fewer benefits of this stimulant even after the use of high doses of caffeine [28]. In this regard, the threshold used to classify individuals as habituated/unhabituated to caffeine greatly varied across investigations [29], which troubles the obtaining of definitive conclusions from these cross-sectional investigations. Up until now, there have been only two investigations that have used a longitudinal research protocol to induce a controlled habituation to caffeine [10,30]. Both investigations agreed on the existence of tolerance to the benefits of caffeine on exercise performance when this substance is ingested chronically in a dose of 3 mg/kg/day for 20 [10] and 28 days [30]. In these two investigations, a progressive tolerance to the ergogenic response of caffeine in comparison to a placebo/control trial was evident, either on the workload attained during a maximal graded exercise test on a cycle ergometer [10] or on the work produced during a 30-min cycling trial. Nevertheless, it is unknown how chronic intake of caffeine may affect submaximal aerobic exercise performed at exercise intensities close to the anaerobic threshold. This information may be valuable to understand how chronic intake of caffeine affects the efficacy of this substance to enhance exercise performance during prolonged activities and it may be useful to plan the use of caffeine in the long-term during endurance training programs. For all these reasons, this study aimed to determine the evolution of tolerance to the ergogenic effect of caffeine on the ventilatory threshold when the substance is ingested for twenty consecutive days. We hypothesized that daily intake of caffeine would progressively reduce the effect of caffeine on the workload attained in the second ventilatory threshold.

## 2. Materials and Methods

Participants: Eleven healthy active individuals (8 men and 3 women) were recruited to participate in this study (mean ± SD: age = 32.3 ± 4.9 years; height = 171 ± 8 cm; body mass = 66.6 ± 13.6 kg; body fat percentage = 16.6 ± 5.0%; and maximal oxygen uptake (VO_2max_) = 48.0 ± 3.8 mL/kg/min). Inclusion criteria were: age between 18 and 40 years; consistent aerobic training (at least 4 days of endurance exercise per week, with at least 45 min of exercise per day); low caffeine consumption (<50 mg of caffeine per day) as defined by Filip et al. [29]; negative smoking status; absence of chronic cardiopulmonary and musculoskeletal diseases; absence of allergy to caffeine and absence of musculoskeletal injuries in the three months prior to the onset of the study. Participants were excluded if they reported medication or dietary supplementation usage within the previous month or any type of menstrual disorders in the sample of female participants. Participants were encouraged to keep a stable endurance fitness level throughout the experiment by keeping their habitual training routines. In addition, participants were informed about the need of avoiding caffeine-containing products and food and any other nutritional supplements for the duration of the study. The willingness to fulfill these standardizations, in addition to the satisfaction of the inclusion/exclusion criteria, was key in the recruitment process of the participants. The week prior to the onset of the experiment, the participants were fully informed of the research procedures, standardizations and the risks associated with the study and gave their informed written consent to participate in the investigation. The study was approved by the Camilo José Cela Institutional Review Board. The required sample size was determined a priori using G*Power software [31]. At least ten participants were required to detect an effect size of 0.66 in oxygen uptake at the workload that produced the ventilatory threshold with the ingestion of caffeine, with a power of 0.80 and the two-tailed α level set at 0.05. This calculation was based on the data provided by Berry et al. when investigating the effect of caffeine on the ventilatory threshold [11].

Experimental design: A longitudinal, double-blind, placebo-controlled, randomized crossover experimental design was used in this study. Each participant took part in two identical treatments and, thus, acted as his/her own control: in one treatment, participants ingested an unidentifiable capsule containing 3 mg of caffeine per kg of body mass for twenty consecutive days; in another treatment, the participants ingested a capsule with the exact same appearance but filled with a placebo (cellulose), which was ingested for the same duration and frequency as in the caffeine treatment. The capsule was ingested daily at 9:00 AM with water and at least 8 h after dinner of the previous day (i.e., in a fasted state). Each twenty-day treatment was identical, except for the substance under investigation: on days 1, 4, 6, 8, 13, 15, 18 and 20 of each treatment, participants ingested the assigned capsule 45 min before performing a maximal ramp exercise test on a cycle ergometer to determine performance variables at the second ventilatory threshold. Each treatment was preceded by a pre-treatment trial (i.e., day 0), aiming to assess the performance variables before the onset of each treatment. On day 0, participants performed the same experimental procedures as in the remaining experimental trials but without ingestion of any capsule. Day 11 also included a measurement of performance, but it was different because the assigned capsule was ingested after—not before—the exercise testing. The experimental procedures on day 11 aimed to have a “control day” at the halfway point of the treatments to aid in identifying differences in the caffeine–placebo comparisons in the subsequent days. All trials were carried out in a laboratory with a dry temperature of 21.3 ± 0.3 °C and a relative humidity of 30 ± 10%. There was a week between treatments to wash-out substances. The results of this protocol on VO_2max_ and maximal wattage during the ramp test have been published elsewhere [10].

Experimental protocol: For the month before the onset of the experiment, participants were encouraged to avoid all sources of dietary caffeine in order to eliminate any habituation to this stimulant. One week before the onset of the first treatment, participants underwent routine medical screening to ensure that they were in good health and suitable for the experiment. Within the week prior to the onset of the experiment, participants were also familiarized twice with all the experimental protocols and body mass was measured (±50 g, Radwag, Radom, Poland) to calculate caffeine dosage. On day 0 of each treatment, participants were provided with a plastic bag containing 20 unidentifiable capsules with the assigned treatment. Participants received a daily remainder through a mobile application to ingest the capsule in the morning. On each experimental day (except for days 0 and 11), participants ingested the capsule in the laboratory and rested for 45 min on a stretcher. On days 0 and 11, participants rested on the stretcher, but this resting period was not preceded by capsule ingestion. Afterwards, participants performed a 10-min standardized warm-up on the cycle ergometer and then performed the ramp exercise test. During the performance test, the initial load was set at 50 watts and it was progressively increased by 25 watts per min until exhaustion. Participants chose their pedaling rate between 70 and 90 rpm, but they had to replicate it in all experimental trials. During the incremental exercise test, pulmonary ventilation, end-tidal partial pressure of oxygen (P_ET_O_2_), oxygen uptake (VO_2_), end-tidal partial pressure of carbon dioxide (P_ET_CO_2_) and carbon dioxide production (VCO_2_) were continuously measured and recorded by means of a breath-by-breath analyzer (Metalyzer 3B, Cortex, Leipzig, Germany). These variables were used to calculate the second ventilatory threshold (VT_2_) by using the ventilatory equivalents for oxygen (VE/VO_2_) and carbon dioxide (VE/VCO_2_) and end-tidal partial pressure changes in accordance with the combined procedure described by Gaskill et al. [32]. Briefly, visual interpretation of each graph was independently performed by two trained researchers who were blinded to the treatments under investigation. These researchers established VT_2_ as the point that elicited an increase in ventilation respect to VO_2_ (i.e., ↑VE/VO_2_) and VCO_2_ (i.e., ↑VE/VCO_2_) while end-tidal partial pressure of carbon dioxide decreased (↓P_ET_CO_2_). With this protocol, the decrement in P_ET_CO_2_, which starts at VT_2_, is evident until the end of the test [33]. A third researcher was sought only in case of disagreement between the two researchers [34]. Once the VT_2_ was agreed, data on wattage, VO_2_, heart rate and VE/VO_2_ and carbon dioxide VE/VCO_2_ at this point were extracted for analysis by using an average of the data for the last 15 s of the workload at VT_2_.

Statistical analysis: Data were analyzed by using the statistical package SPSS v 20.0 for statistical analysis. Initially, the existence of normal distribution was tested for each variable with the Shapiro–Wilk test. All the variables presented a normal distribution and parametric statistics were used afterwards. Additionally, the sphericity assumption was checked with Mauchly’s test. If this assumption presented a probability of *p* < 0.05, the Greenhouse–Geisser correction was used. We performed a two-way analysis of variance (ANOVA) with repeated measures (substance × time) in each variable to determine the differences in the caffeine–placebo comparison on each day of treatment and differences in respect to day 0 within each treatment. In the case of a significant F value in the ANOVAs, the differences between groups were identified with LSD post-hoc tests. The significance level was set at *p* < 0.05. The effect size ± 95% confidence intervals (CI) was also calculated in all caffeine–placebo comparisons on the same day of treatment by using Cohen’s *d* units [35]. The magnitude of the effect size (ES) was interpreted as follows: less than 0.2 = trivial; between 0.2 and 0.6 = small; between 0.6 and 1.2 = moderate; between 1.2 and 2.0 = large; between 2.0 and 4.0 = very large; higher than 4.0 = extremely large [36].

## 3. Results

**Figure 1:**
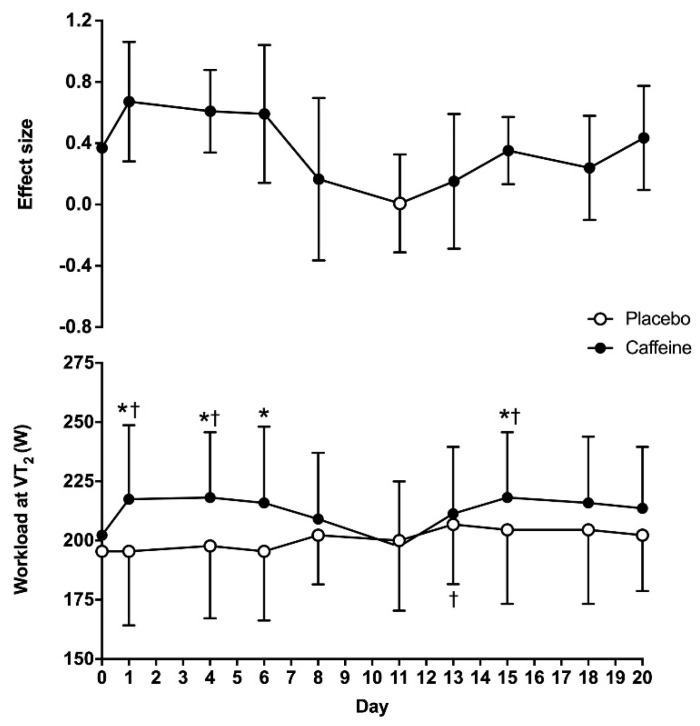
Workload at the second ventilatory threshold (VT_2_) during a cycling ramp test with the administration of 3 mg/kg/day of caffeine or a placebo for 20 consecutive days. The upper panel shows effect sizes (±95% confidence intervals) for all timepoint comparisons between caffeine and the placebo. The lower panel depicts data presented as mean ± standard deviation. (*) Caffeine different from placebo for the same timepoint at *p* < 0.05. (†) Different from day 0 within the same treatment at *p* < 0.05.

The two-way ANOVA revealed statistically significant main effects of substance (F = 26.11; *p* < 0.01) and time (F = 1.99; *p* = 0.04) on the wattage obtained at VT_2_, although there was no interaction between these two factors (F = 1.73; *p* = 0.09). On day 0, the wattage obtained at VT_2_ was similar between the placebo and caffeine (195 ± 27 vs. 202 ± 18 W, respectively; *p* = 0.27). In the 20-day placebo treatment, the wattage at VT_2_ was higher on day 13 when compared to day 0 (*p* = 0.02), with no other differences in respect to day 0. In the 20-day caffeine treatment, the wattage at VT_2_ was higher on days 1, 4 and 15 than on day 0 (*p* < 0.05). In addition, the workload attained at VT_2_ was higher with caffeine than with the placebo on days 1, 4, 6 and 15 (*p* < 0.05). The ES of the caffeine–placebo comparison was moderate on days 1, 4 and 6 and it decreased to small afterwards (Figure 1).

**Figure 2:**
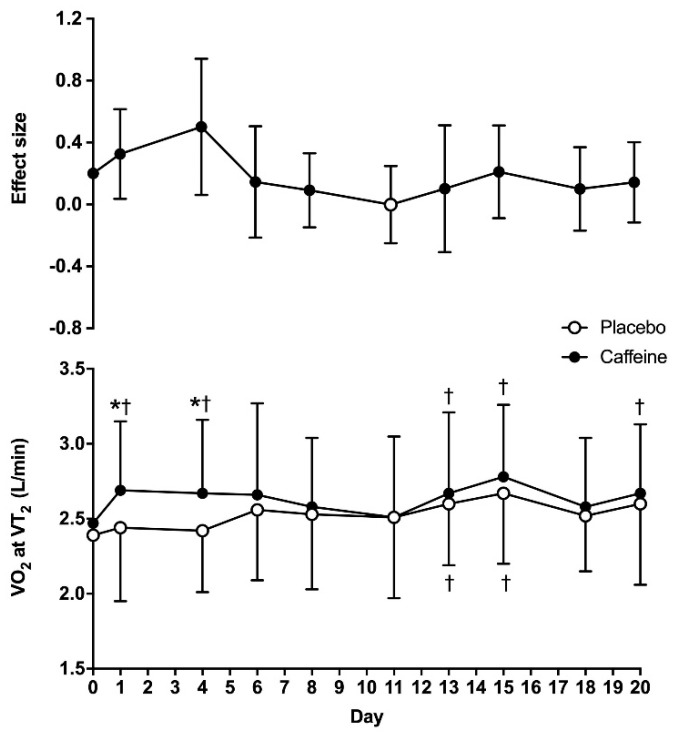
Oxygen uptake (VO_2_) at the second ventilatory threshold (VT_2_) during a cycling ramp test with the administration of 3 mg/kg/day of caffeine or placebo for 20 consecutive days. The upper panel shows effect sizes (±95% confidence intervals) for all timepoint comparisons between caffeine and a placebo. The lower panel depicts data presented as mean ± standard deviation. (*) Caffeine different from placebo for the same timepoint at *p* < 0.05. (†) Different from day 0 within the same treatment at *p* < 0.05.

For VO_2_ obtained at VT_2_, there were main effects of substance (F = 6.65; *p* = 0.03) and time (F = 3.25; *p* = 0.16), with no interaction between these two factors (F = 0.95; *p* = 0.49). The VO_2_ obtained at VT_2_ on day 0 was similar between the placebo and caffeine (2.39 ± 0.43 vs. 2.47 ± 0.44 L/min, respectively; *p* = 0.31). In the placebo treatment, VO_2_ at VT_2_ was higher on days 13 and 15 when compared to day 0 (*p* < 0.05), with no other differences in this treatment. In the caffeine treatment, VO_2_ at VT_2_ was higher on days 1, 4, 13, 15 and 20 than on day 0 (*p* < 0.05). In addition, VO_2_ at VT_2_ was higher with caffeine than with the placebo on days 1 and 4 (*p* < 0.05). The ES of the caffeine–placebo comparison was small on days 1 and 4 and became trivial afterwards (Figure 2).

**Figure 3:**
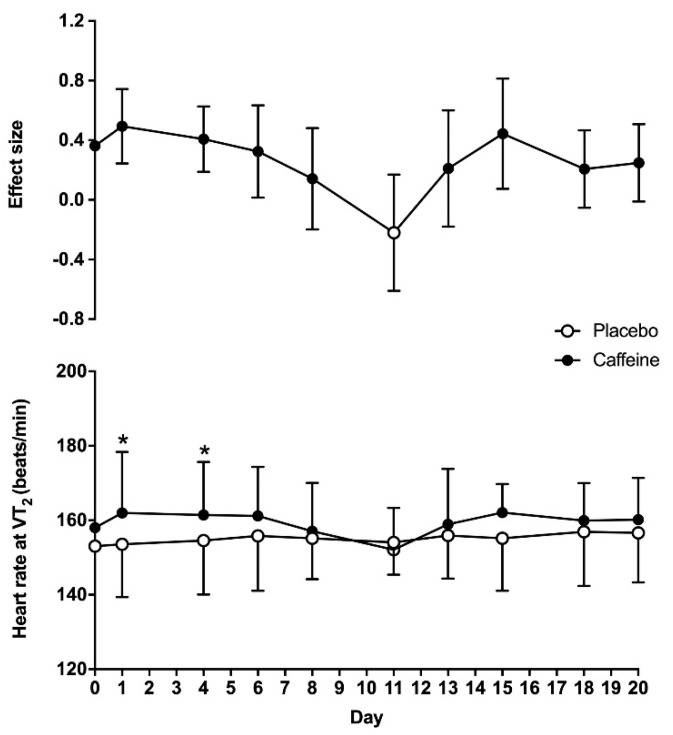
Heart rate at the second ventilatory threshold (VT_2_) during a cycling ramp test with the administration of 3 mg/kg/day of caffeine or a placebo for 20 consecutive days. The upper panel shows effect sizes (±95% confidence intervals) for all timepoint comparisons between caffeine and the placebo. The lower panel depicts data presented as mean ± standard deviation. (*) Caffeine different from placebo for the same timepoint at *p* < 0.05.

For heart rate obtained at VT_2_, there was a main effect of substance (F = 18.5; *p* < 0.01), while the overall effect of time (F = 1.93; *p* = 0.13) and the interaction (F = 1.51; *p* = 0.22) did not reach statistical significance. On day 0, heart rate at VT_2_ was similar between the placebo and caffeine treatments (153 ± 14 vs. 158 ± 16 beats/min, respectively; *p* = 0.15). Heart rate at VT_2_ was higher with caffeine than with the placebo on days 1 and 4 (*p* < 0.05). The ES of the caffeine–placebo comparison was small on days 1, 4 and 6 and became trivial afterwards (Figure 3).

There was a main effect of substance (F = 9.73; *p* = 0.01) on the ventilatory equivalent of O_2_ at VT_2_, while the overall effect of time (F = 1.90; *p* = 0.12) and the interaction between the two (F = 0.76; *p* = 0.65) did not reach statistical significance for this variable. The ventilatory equivalent of O_2_ at VT_2_ was similar between the placebo and caffeine on day 0 (Table 1, *p* = 0.17). The ventilatory equivalent of O_2_ at VT_2_ was higher on days 4, 6, 13 and 15 with caffeine than with the placebo (*p* < 0.05). Regarding the ventilatory equivalent of CO_2_ at VT_2_, there was a main effect of substance (F = 23.6; *p* < 0.01), while the overall effect of time (F = 1.23; *p* = 0.28) and the interaction between the two (F = 0.72; *p* = 0.68) did not reach statistical significance. The ventilatory equivalent of CO_2_ at VT_2_ was similar between the placebo and caffeine on day 0 (*p*= 0.09) but it was higher with caffeine than with the placebo on day 1 (*p* = 0.04).

## 4. Discussion

The transition point indicating that the obtaining energy within skeletal muscle changes from aerobic pathways to a mixed model of aerobic-plus-anaerobic metabolism is one of the most important physiological variables for endurance sports. In this regard, the exercise intensity that enables maximum production of energy through aerobic pathways, without a concomitant accumulation of metabolites from anaerobic metabolism, is a key parameter with several practicalities, such as monitoring the efficacy of endurance training, estimating endurance performance and designing training programs to achieve specific adaptations [37]. Although there are several methods to assess this metabolic transition point, the use of pulmonary ventilatory thresholds during incremental intensity exercise is one of the most accurate methods to estimate the exercise intensity that produces the anerobic threshold [38]. Interestingly, evidence clearly indicates that acute caffeine intake increases endurance performance [4,5,6,7], but the effect of caffeine on physiological variables attained at pulmonary thresholds has been less well investigated [11], and the ergogenic effect of caffeine at this point is even less clear [39]. In addition, there is no previous investigation that has determined whether the potential effect of caffeine intake on increasing the exercise intensity at the ventilatory threshold is maintained when caffeine is ingested chronically. The resolution of these two topics is key for those endurance athletes using caffeine or caffeine-containing supplements before training and competitions, as caffeine may modify the exercise intensity at the anaerobic threshold, while its chronic use may progressively reduce the potential ergogenic benefit of caffeine due to habituation to this substance. With these motivations, the purpose of this study was to determine the time course of tolerance to the ergogenic effect of caffeine on the second ventilatory threshold during a cycling ramp test when the substance is ingested for twenty consecutive days. To allow a clearer picture of the habituation to the ergogenic effect of caffeine, we used a double statistical approach with *p* values and effect sizes. The main outcomes of this study are as follows: (a) on the first day of ingestion, pre-exercise ingestion of 3 mg caffeine/kg body mass increased the workload at VT_2_ by 7.7 ± 9.7% when compared to the ingestion of a placebo. This acute ergogenic effect was associated to increases in VO_2_ and heart rate at VT_2_. (b) There was a main effect of caffeine on the workload attained at VT_2_. However, in the caffeine–placebo comparison, within the same day of treatment, the effect of caffeine to increase the exercise workload at VT_2_ over the placebo was only present for the first two weeks of ingestion (except for day 8 and day 13), while the size of the benefit of caffeine on this performance variable decreased progressively over time (Figure 1). (c) There was a main effect of caffeine on heart rate and VO_2_ at VT_2_, but the caffeine–placebo pairwise comparisons revealed that caffeine did not produce any statistically significant difference over the placebo on these variables after 4 days of consecutive ingestion (Figure 2 and Figure 3). Additionally, there was a progressive reduction in the influence of caffeine on VO_2_ and heart rate at VT_2_ with chronic ingestion. Taken together, this information suggests that acute caffeine intake has the capacity to increase the absolute workload attained at VT_2_ during a cycling ramp test, which may explain the performance-enhancing effect of this stimulant during aerobic exercise of submaximal intensity. However, it seems apparent that the ergogenic effect of caffeine progressively diminished with chronic ingestion, and it was not present, in statistically significant terms, after 15 days of consecutive ingestion. As practical information, endurance athletes seeking to obtain performance-enhancing effects of caffeine during endurance training or competition should avoid habituation to caffeine, as the existence of a gradual tolerance to caffeine’s ergogenicity on VT_2_ seems clear.

Acute caffeine intake has the potential to increase oxygen uptake at the ventilatory threshold (7 mg/kg, [11]); reduces the accumulation of blood lactate for the same exercise intensity (10 and 15 mg/kg, [12]); increases time to exhaustion at an intensity 10% below the anaerobic threshold (5 mg/kg, [40]); augments the ventilatory response for a given amount of CO_2_ produced during exercise (650 mg of caffeine [41]). All these findings point towards an ergogenic benefit of acute caffeine consumption to enhance performance when exercising at an intensity close to the anaerobic threshold. However, to our knowledge, this is the first investigation that finds an increase in the exercise intensity that enables VT_2_ with the ingestion of a moderate dose of caffeine (i.e., 3 mg of caffeine per kg of body mass). The obtaining of a higher absolute workload at VT_2_ may be the result of different physiological responses produced by acute caffeine intake. First, the blockade of adenosine receptors due to caffeine may produce a higher neuromuscular activation during exercise, ultimately affecting performance [42]. In addition, the blockade of adenosine receptors also increases the liberation of dopamine, norepinephrine and serotonin, among other neurotransmitters, which may allow lower perceived fatigue during exercise [43]. Caffeine also produces a respirogenic effect that augments ventilation during exercise of submaximal intensity [39,44], which may allow a higher maintenance of muscle pH through the buffering of H^+^ and the elimination of non-metabolic CO_2_ by respiration [45]. This mechanism was found in the current investigation as the ventilatory equivalent of CO_2_ at VT_2_ with caffeine was higher than with the placebo (Table 1). Finally, better muscle oxygenation during aerobic exercise of submaximal intensity may delay the use of anaerobic metabolism during incremental exercise [16], allowing higher exercise intensity at the aerobic–anaerobic transition point. All this evidence supports that acute caffeine intake increases the workload at VT_2_, suggesting a potential benefit of this stimulant for aerobic exercise performed close to the anaerobic threshold.

There was a main effect of caffeine on wattage, heart rate and VO_2_ attained at VT_2_, which indicates that this substance was effective in modifying the point at which the second ventilatory threshold is obtained during a ramp cycling test. Interestingly, there was no interaction between substance × time in any of these three variables, which may indicate that the effect of caffeine over the placebo was not affected by the duration of the treatment. However, the caffeine–placebo pairwise comparison indicates that the performance benefit of caffeine on VT_2_ was obtained on several days for the first two weeks of chronic caffeine administration, but it disappeared afterwards. Furthermore, the size of the ergogenic benefit of caffeine on VT_2_ gradually reduced with long-term ingestion, at least up to twenty days of daily administration. This confirms the existence of tolerance to the ergogenic benefit of caffeine for aerobic exercise of submaximal intensity, as it has been found in aerobic exercise of maximal intensity (i.e., exercise at VO_2max_) [10] and in the maximum amount of work produced during 30 min of exercise [30]. Habituation to caffeine and subsequent tolerance to the benefits of this stimulant on exercise have been long speculated [46], but evidence with well-controlled investigations has been only recently provided. With the current evidence, it seems clearer that chronic ingestion of caffeine, at a dose of 3 mg/kg/day, produces tolerance to some of the benefits of caffeine on exercise, at least during laboratory testing. Interestingly, caffeine intake was still able to produce a non-significant but measurable effect on the workload attained at VT_2_ (Figure 1) and at VO_2max_ [10] after twenty days of consecutive ingestion. This may indicate that caffeine intake still produced small benefits after twenty days of ingestion, although the magnitude of this effect was minor. Future investigations with longer periods of chronic caffeine ingestion are needed to unveil whether caffeine ergogenicity disappears with daily ingestion for >20 days. In addition, it is also necessary to investigate whether acute ingestion of a higher dose of caffeine (i.e., >3 mg/kg) in habituated individuals may offset the tolerance developed with the ingestion of 3 mg/kg/day.

The present research has different limitations that should be discussed. First, VT_2_ was identified using a visual interpretation of the relationships of VE/VO_2_, VE/VCO_2_, P_ET_O_2_, and P_ET_CO_2_ with exercise intensity. Although this is a valid methodology [32], this method is subject to human error and interpretation. To reduce this error, two trained researchers independently established VT_2_ at each day of caffeine/placebo ingestion without any knowledge about the individuals or the substances under investigation. In most cases, they agreed in their visual identification of VT_2_ and the seeking of a third researcher to solve disagreements was anecdotical. Second, we used VT_2_ as an estimate of the anaerobic threshold during exercise of increasing intensity. The use of blood samples to assess lactate concentration would be helpful to improve the identification of the aerobic–anaerobic transition point. Finally, we used a moderate dose of caffeine to induce tolerance and the time of chronic ingestion was relatively short. Therefore, the results of the current investigation may not apply to endurance athletes with higher daily caffeine ingestion or those using caffeine chronically for several months/years. Despite these limitations, the current investigation may be helpful for athletes and coaches when planning the use of caffeine during endurance training and competition.

## 5. Conclusions

The acute intake of caffeine (i.e., day 1 of ingestion) at a dose of 3 mg per kg of body mass, prior to exercise, increased the workload attained at VT_2_ with additional and concomitant benefits on VO_2_ and heart rate at VT_2_. In addition, there was a main effect of caffeine on the workload, VO_2_ and heart rate attained at VT_2_ when the substance was ingested chronically for twenty days, with no substance × time interaction. However, the magnitude of the ergogenic response of caffeine progressively attenuated with the daily ingestion of this substance for twenty days. This is because caffeine–placebo pairwise comparisons revealed that caffeine was only effective to enhance the exercise workload at VT_2_ for the first two weeks of chronic caffeine administration, but this benefit disappeared afterwards. Regarding VO_2_ and heart rate at VT_2_, the benefits of caffeine were only present for the first four days of consecutive administration. From a practical perspective, a dose of 3 mg of caffeine per kg of body mass may be acutely employed to enhance exercise intensity at the second ventilatory threshold, which may represent a potential benefit for training and competition. However, the progressive tolerance to the ergogenic benefit of caffeine on VT_2_ suggests the convenience of avoiding daily ingestion of caffeine. Although scarce, there is evidence suggesting that daily ingestion of caffeine for 8 weeks, in conjunction with aerobic training, does not provided further benefits than aerobic training without supplementation [47]. In addition, daily ingestion of caffeine for twenty days may produce a progressive rise in some adverse effects associated to caffeine intake, such as increased nervousness and vigor, irritability, insomnia and diuresis [48]. Perhaps the use of caffeine only before a competition and/or before high-intensity training may be an acceptable option to maximize the benefits of this stimulant while reducing tolerance and side effects.

## Figures and Tables

**Table 1:** Ventilatory equivalent for oxygen (O_2_) and for carbon dioxide (CO_2_) at the second ventilatory threshold (VT_2_) during a cycling ramp test with the administration of 3 mg of caffeine per kg of body mass per day or a placebo for twenty consecutive days.

	Ventilatory Equivalent of O_2_ at VT_2_			Ventilatory Equivalent of CO_2_ at VT_2_		
Day	Placebo	Caffeine	Effect Size (±95% CI)	Placebo	Caffeine	Effect Size (±95% CI)
0	28.2 ± 3.2	29.9 ± 3.5	0.5 (−0.2/1.1)	24.4 ± 2.1	25.8 ± 2.2	0.4 (−0.1/1.0)
1	29.8 ± 4.8	30.6 ± 3.9	0.2 (−0.4/0.7)	25.1 ± 2.6	27.2 ± 3.7 *	0.7 (0.2/1.2)
4	29.0 ± 2.5	32.8 ± 4.1 *	1.3 (0.5/2.1)	25.1 ± 2.0	26.0 ± 2.4	0.4 (−0.3/1.1)
6	28.1 ± 2.4	30.6 ± 2.4 *	0.9 (0.3/1.4)	25.7 ± 1.9	26.6 ± 2.7	0.4 (−0.3/1.1)
8	28.6 ± 1.8	30.6 ± 4.5	0.9 (−0.2/2.1)	25.0 ± 1.4	26.0 ± 2.4	0.6 (−0.4/1.6)
11	28.3 ± 2.3	29.3 ± 2.8	0.4 (0.0/0.7)	25.1 ± 1.9	25.4 ± 1.5	0.1 (−0.5/0.8)
13	28.2 ± 2.2	30.5 ± 2.6 *	0.9 (0.5/1.3)	25.7 ± 1.5	26.6 ± 1.7	0.5 (−0.1/1.1)
15	28.0 ± 3.1	30.9 ± 2.5 *	0.8 (0.2/1.4)	26.5 ± 2.6	26.7 ± 2.1	0.1 (−0.2/0.4)
18	29.9 ± 2.8	32.2 ± 3.8	0.7 (−0.1/1.4)	25.8 ± 2.0	26.0 ± 2.5	0.0 (−0.4/0.4)
20	29.0 ± 3.7	31.2 ± 3.4	0.6 (−0.2/1.1)	26.4 ± 2.2 †	26.2 ± 2.0	0.1 (−0.7/0.6)

CI = confidence interval; (*) the value in the caffeine trial was different to the value in the placebo trial within the same day of intervention (*p* < 0.05). The effect size was calculated in all caffeine–placebo comparisons within the same day of intervention.
